# The effect of resistance exercise on multimodal pain thresholds in local and systemic muscle sites

**DOI:** 10.14814/phy2.16123

**Published:** 2024-06-18

**Authors:** Kaitlyn M. Lyons, Matt S. Stock, William J. Hanney, Abigail W. Anderson

**Affiliations:** ^1^ School of Kinesiology and Rehabilitation Sciences, College of Health Professions and Sciences University of Central Florida Orlando Florida USA

**Keywords:** exercise induced hypoalgesia, heat pain threshold, pressure pain threshold, quantitative sensory testing, resistance exercise

## Abstract

Dynamic resistance exercise may produce reductions in pain locally at the exercising muscle and systemically at non‐exercising sites. However, limited research has examined these changes with multiple noxious stimuli. This study examined changes in heat pain threshold (HPT) and pressure pain threshold (PPT) on different musculature after an upper and lower body exercise to compare local and systemic effects. A crossover design with 28 participants (mean age: 21 ± 4 years, 21 female) completed three sessions. Visit one included baseline quantitative sensory testing and 5‐repetition maximum (RM) testing for upper (shoulder press) and lower (leg extension) body. In subsequent sessions, participants performed upper or lower body exercises using an estimated 75% 1‐RM with pre/post assessment of HPT and PPT at three sites: deltoid, quadriceps, and low back. A significant three‐way interaction was observed for HPT (F (1.71, 3.80) = 2.19, *p* = 0.036, *η* ^2^ *p* = 0.12) with significant increases in HPT over the quadriceps (*p* = 0.043) after leg extension and over the deltoid (*p* = 0.02) after shoulder press. Significant systemic changes were not observed for HPT or PPT. Local but not systemic effects were demonstrated after an acute bout of exercise. Peripheral pain sensitivity may be more responsive to heat stimuli after resistance exercise.

## 1 INTRODUCTION

It is widely accepted that regular engagement in resistance exercise significantly enhances muscular strength and hypertrophy, contributing to improved physical performance in healthy individuals. (Fundamentals of Resistance Training: Progression and Exercise Prescription—PubMed, 2023; Schoenfeld et al., 2016; 2017; Westcott, 2012) Due to these benefits, as well as the positive effect on pain, resistance exercise is frequently incorporated as a therapeutic intervention in orthopedic rehabilitation. (Chen et al., 2021; Turner et al., 2020) Resistance exercise produces an acute reduction in pain, referred to as exercise‐induced hypoalgesia (EIH), measured by a decrease in clinical pain intensity or an increase in pain threshold following exercise. (Naugle et al., 2012; Vaegter & Jones, 2020; Wewege & Jones, 2021).

Previous research suggests isometric resistance exercise induces small to moderate EIH responses in healthy individuals. (Naugle et al., 2012; Wewege & Jones, 2021) However, a paucity of evidence exists to evaluate the effect of EIH during dynamic resistance exercise, a common exercise modality in rehabilitation. Notably, a systematic review with meta‐analysis published in 2012, encompassing studies on EIH effects in healthy individuals and those with pain, found only two studies that incorporated dynamic resistance exercise, with pressure pain threshold (PPT) measured at a systemic site, the forefinger, in both studies. (Focht & Koltyn, 2009; Koltyn & Arbogast, 1998; Naugle et al., 2012) A more recent systematic review published in 2021 included 13 studies but only 3 utilized dynamic resistance exercise as an intervention. (Wewege & Jones, 2021) Collectively, the body of literature supports that dynamic resistance exercise produces increases in pain threshold. However, gaps remain, highlighting the need for further investigation, particularly regarding the specific muscular sites affected by dynamic resistance exercise.

A method to examine local and systemic effects of dynamic resistance exercise is to directly compare pain thresholds at multiple sites during an upper and lower body resistance exercise. In the 2021 systematic review, of the three studies mentioned previously, none compared the effects of a single bout of upper body to lower body dynamic resistance exercise, and to our knowledge, this relationship has not been directly compared. (Wewege & Jones, 2021) This gap in the literature prompted the need to investigate potential local and whole‐body effects of a single bout of upper and lower body resistance exercise on pain sensitivity. This could better inform clinicians and rehabilitation specialists on exercise interventions aimed at targeting local and systemic pain.

Additionally, limited studies have used a multi‐modal pain assessment (both heat and pressure) to assess EIH. In a 2020 literature review (Vaegter & Jones, 2020), only 9 out of 50 studies investigating isometric or dynamic resistance exercise included measures of heat and pressure. Within these studies, conflicting findings were demonstrated regarding heat testing specifically. Several studies indicated there was a decrease in pain ratings during heat testing, but none specifically reported heat pain threshold (HPT). (Bishop et al., 2011; Crombie et al., 2018; Harris et al., 2018; Jones et al., 2016; Koltyn et al., 2014; Naugle et al., 2014, 2016; Ohlman et al., 2018; Vaegter et al., 2017) Including multiple measures of pain is important since these behaviorally reflect different underlying mechanisms of pain. A‐delta fibers are primarily stimulated during mechanical pressure detection and vibration and C‐fibers are the primary nerve fibers stimulated for HPT. A‐delta‐fibers are deep tissue myelinated, fast‐conducting fibers that provide precise localization of pain, while C‐fibers are more superficial; unmyelinated, slow‐conducting fibers that respond to various high‐intensity stimuli. (Pain Principles (Section 2, Chapter 6) Neuroscience Online: An Electronic Textbook for the Neurosciences | Department of Neurobiology and Anatomy—The University of Texas Medical School at Houston, 2024). Including a multi‐modal assessment of pain during EIH may provide a more comprehensive understanding of the neurophysiologic underpinnings of changes in pain during exercise.

Without a direct comparison of upper and lower body exercise plus multi modal assessment of pain at multiple sites, the local and systemic effects remain unclear. Therefore, the primary aim of this study was to compare the immediate changes in PPT by testing a local and systemic site between upper and lower body resistance exercise. We hypothesized PPT pain threshold would have a greater increase at the local exercising muscle after a single bout of lower and upper body resistance exercise. The secondary aim was to compare the immediate changes in HPT by testing sites between upper and lower body resistance exercise. We hypothesized that HPT would exhibit a greater increase at the local exercising muscle after a single bout of lower and upper body resistance exercise.

## 2 METHODS

### 2.1 Participants

Thirty healthy participants between the ages of 18–60 years who were not currently experiencing pain completed the study. This age range was chosen to obtain a broad representation of individuals for this study. Participants were excluded for the following reasons: inability to appropriately perform the intervention, not English speaking, regularly using prescription pain medications, current or history of a chronic pain condition, use of blood‐thinning medication, presence of a medical condition known to affect sensation, contraindication to the application of ice (including blood pressure >140/90 mmHg), known presence of cardiovascular, pulmonary, or metabolic disease, currently using tobacco products, not physically ready to exercise without a medical exam as indicated by the Physical Activity Readiness Questionnaire Plus (PAR‐Q+), surgery, injury, or fracture within the past 6 months, or pregnant. Participants were not given specific instructions to follow before any session, normal activities of daily living were to be continued.

The University of Central Florida's Institutional Review Board (IRB) approved this study (Study #5752) and all participants provided written informed consent. The study was prospectively registered with clinicaltrials.gov (NCT05985382).

### 2.2 Study overview

**FIGURE 1:**
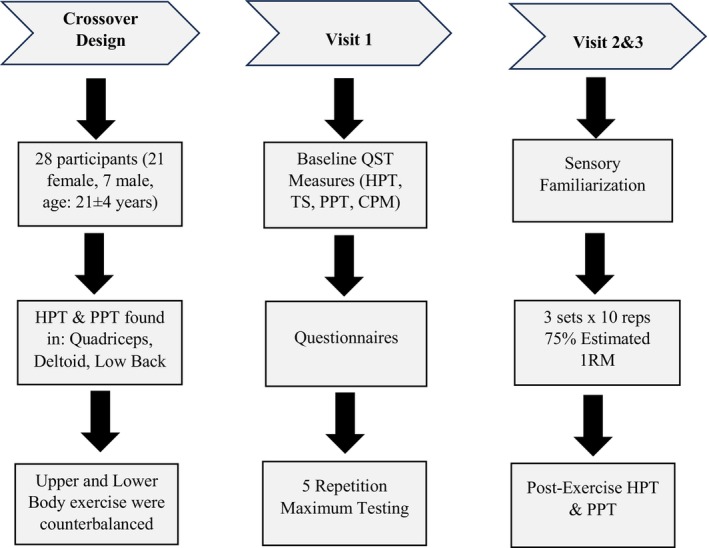
Study overview. CPM, conditioned pain modulation; HPT, heat pain threshold; PPT, pressure pain threshold; TS, temporal summation.

As demonstrated in Figure 1, participants attended three testing sessions separated by a minimum of 48 h: (Session 1) Informed Consent and Baseline Testing, (Sessions 2 and 3) Experimental Sessions consisting of either upper (shoulder press) or lower body (leg extension) resistance exercise completed for 3 sets, 10 repetitions administered in a counterbalanced order among participants.

#### 2.2.1 Session 1: Baseline testing

Participants reported their age, sex, race, ethnicity, height, weight, exercise minutes per week with a standard demographic questionnaire. Next, participants were familiarized with quantitative sensory testing.

### 2.3 Quantitative sensory testing

Quantitative sensory testing encompasses a battery of assessments designed to gauge perceptual responses to systematically administered and quantifiable sensory stimuli, typically involving pressure, thermal, or electrical stimuli. These tests commonly involve the evaluation of pain threshold, the minimum stimulus intensity at which an individual perceives pain.

Testing was performed in the following order: HPT, temporal summation, PPT, and then conditioned pain modulation. Participants rated pain during these tests with a 101‐point numeric pain rating scale (NPRS) in which 0 indicated “no pain” and 100 indicated the “the most intense pain sensation imaginable.” The NPRS demonstrates sound psychometric properties in rating pain. (Bolton & Wilkinson, 1998; Hartrick et al., 2003; Jensen et al., 1999) Heat and PPT were performed during the baseline testing session to familiarize participants with these measures prior to the second and third session. All quantitative sensory testing was completed by the same investigator during each visit to maintain consistency for each measure.

### 2.4 Heat pain threshold

Thermal Stimuli were delivered with the TCS II from QST.Lab (Strasbourg, France) with a T11 probe that has a total summation surface of 6.6 × 4 × 4.4 cm (l × w × h). The thermode increased at 1°C/s from a baseline temperature of 32°C to a maximum temperature of 50°C. Participants were instructed to press a button when the sensation changed from “comfortable warmth to slightly unpleasant pain.” This was applied to the dominant quadriceps (halfway between the hip crease and the superior border of the patella), deltoid (middle of the muscle belly, halfway between the acromion and the insertion of the deltoid), and low back (dominant erector located 2 cm above the PSIS). (Jones et al., 2016; Kennedy et al., 2016; Kosek & Ekholm, 1995; Price et al., 2002; Vaegter & Graven‐Nielsen, 2016; Walton et al., 2014). Pain at threshold was rated from 0 to 100, using the NPRS. The average temperature and pain ratings of two trials were analyzed, with 30 s between each trial (Moloney et al., 2012).

### 2.5 Temporal summation (TS)

TS is a suprathreshold heat pain stimuli that occurs when repetitive C‐fiber input results in increased output from the dorsal horn neurons, referred to as “wind up.” To examine this, the thermode was applied to the palm of the non‐dominant hand. Ten heat pulses with a standard inter‐stimulus interval and a peak of 49°C were applied. (Wewege & Jones, 2021) The NPRS was used by participants to rate the sensation of “second pain” felt during each heat pulse. Second pain is indicative of pain facilitatory response and is believed to be primarily mediated by C‐fibers. TS was calculated as: NPRS on the fifth pulse‐NPRS on the first pulse. (Valencia et al., 2011).

### 2.6 Pressure pain testing

A computerized pressure algometer (AlgoMed, Ramat Yishai, Israel) with a 1 cm rubber tip was applied at a constant rate. Pressure was gradually increased until the sensation changed from “comfortable pressure to slightly unpleasant pain.” When PPT was reached, the participant pressed a response button. (Wewege & Jones, 2021) For PPT testing, two trials were performed on the dominant quadriceps, deltoid, and low back, with 30 s between each trial. Participants were seated with their legs supported during the quadriceps and deltoid trials and prone on a table during the low back trial. Participants rated pain at threshold with the 101‐point NPRS. The mean pressure and pain rating scores were analyzed for each site. For safety, pressure did not exceed 1000 kilopascals (kPa).

### 2.7 Conditioned pain modulation (CPM)

CPM is a behavioral measure of pain inhibitory process in which the secondary application of a painful stimuli inhibits pain at a primary site. (Yarnitsky et al., 2015) To test this, first, PPT was applied to the webspace of the non‐dominant foot for two trials. Next, participants submerged their dominant hand into a refrigeration unit (ARCTIC Series Refrigerated Bath Circulator, ThermoFisher Scientific, Massachusetts, USA) that maintained a constant water temperature of 12°C for 1 min. Participants rated their cold pain every 15 s using the NPRS. Finally, once the hand was removed from the water, the PPT was repeated on the web space of the non‐dominant foot. A washout period of 15 min was completed before exercise testing, allowing for inhibitory effects to stabilize. (Kennedy et al., 2016) CPM was calculated as the first PPT minus the last PPT so results denoting pain inhibition are reported as a negative value. (Yarnitsky et al., 2015).

### 2.8 Five repetition maximum (5‐RM)

The 5‐RM testing protocol was performed using the National Strength and Conditioning Association (NSCA) protocol. (Beachle, 2016) The 5‐RM test was selected as it is a suitable alternative to the 1‐RM test and not as strongly influenced by participant training status. (Gail & Künzell, 2014) 5‐RM was conducted for the lower body (leg extension machine [Star Trac Leg Extension]) and upper body (shoulder press [Star Trac Shoulder Press]). Consistent with NSCA protocol, warm‐up sets of 10, 8, and 6 repetitions were completed for each exercise with 3–4 min of rest between each set. (Beachle, 2016) The general guidelines for repetition tempo were followed, a 2–4 s eccentric and a 1–2 s concentric were deemed acceptable by the NSCA. A certified strength and conditioning professional was present for all sessions and verbally cued participants if a proper tempo or technique was not being used at any point for each exercise. (Beachle, 2016) This weight was used to estimate the 1‐RM using a pre‐determined equation: (Weight × Reps × 0.333) + Weight, established by the National Strength and conditioning association, (Beachle, 2016; Epley, 1985) 75% of the estimated 1‐RM was used to complete the exercises performed in Sessions 2 and 3.

#### 2.8.1 Sessions 2 and 3

Visits for sessions two and three were scheduled with at least 48 h apart, at approximately the same time of day, with no more than 1 week between each visit. Participants were informed after arrival on the type of exercise, upper or lower body, that would be completed. The investigator was not blinded to the intervention being performed. Participants performed an individualized warm‐up set of 10 repetitions at a weight approximately 50% lighter than the weight calculated for the exercise session. Participants then completed either the upper body exercise (shoulder press) or lower body exercise (leg extension) during Sessions 2 and 3 with the order counterbalanced among participants. Participants were asked to verbally rate their fatigue after each set using the 0–10 Borg scale, 2–3 min of rest were given in between each set of the exercise.

Two trials, with 30 s between each trial, for HPT were performed on the dominant quadriceps, deltoid, and low back immediately before and after each exercise per the previously described protocol. For each testing visit, HPT was performed before PPT to minimize cross‐modal interference and potential sensitization or desensitization of sensory receptors or pathways. Additionally, each muscle site was tested in the same order as previously listed to ensure consistency. The NPRS was used to rate pain for each trial of heat and pressure pain testing. For the upper body exercise, HPT and PPT were applied to the deltoid to examine a “local” EIH response while the low back and quadriceps examined a “distant” or “systemic” EIH response. The deltoid was chosen as it is a consistent measure used in QST testing in healthy individuals. (Bilika et al., 2023) For the lower body exercise, HPT and PPT applied to the quadriceps examined a “local” EIH response while the low back and deltoid examined a “distant” or “systemic” EIH response. (Jones et al., 2016; Vaegter & Graven‐Nielsen, 2016) These testing sites were consistent with prior studies implementing isometric quadriceps exercise. (Jones et al., 2016b; Vaegter & Graven‐Nielsen, 2016) The low back was chosen as it is a site that is systemic to both exercising areas and provides a systematic approach of ensuring the same non‐exercising site is measured for both upper and lower body exercise.

To standardize the application site, quadriceps stimuli were applied halfway between the hip crease and superior border of the patella. The low back stimuli were applied on the dominant erector located approximately 5 cm above the posterior superior iliac spine. The deltoid stimulus was applied in the middle of the muscle belly, halfway between the acromion and the insertion of the deltoid. All testing sites were applied to the dominant side of the body.

### 2.9 Lower body resistance exercise

Participants performed a quadriceps contraction into terminal knee extension and eccentrically returned to the resting position in flexion at 75% of the estimated 1‐RM using the leg extension machine (Star Trac Leg Extension, Flex Fitness Inc., San Bernadino, CA). The research assistant ensured proper technique and compensatory movements, such as trunk flexion, were avoided. Three sets of 10 repetitions were completed with 3–4 min of rest between each set. (Focht & Koltyn, 2009; Koltyn & Arbogast, 1998).

### 2.10 Upper body resistance exercise

Participants performed a shoulder press with a weight equivalent to 75% of the estimated 1‐RM for 3 sets of 10 repetitions on the Star Trac shoulder press machine (Flex Fitness Inc., San Bernadino, CA). A 3–4 min rest was provided between each set. Participants contracted the deltoid into overhead shoulder extension and the research assistant ensured compensatory movements such as low back extension were avoided. (Focht & Koltyn, 2009; Koltyn & Arbogast, 1998).

### 2.11 Statistical analysis

JASP version 0.17.3 (University of Amsterdam, Netherlands) was used for statistical analyses. Descriptive statistics were calculated to characterize the total sample by demographic and pain sensitivity factors. All values are presented as mean (standard deviation [SD]).

The primary purpose of the study was to examine the effects of upper and lower body resistance exercise on heat and PPTs by testing site. A three‐way repeated measures analysis of variance (ANOVA) examined for a time (pre/post) × site (deltoid, quadriceps, low back) × condition (upper, lower body) effect. Simple effects decomposition with Bonferroni correction were performed. Separate analyses were performed for PPT and HPT. Mauchly's test of sphericity was performed and, if the assumption of sphericity was violated the Greenhouse–Geisser correction was applied. An alpha level of 0.05 was established.

As an additional exploratory analysis, a Pearson correlation examined for the association between baseline pain sensitivity and EIH response during upper and lower body exercise. EIH change score was calculated as: average post‐exercise PPT (or HPT)‐ average pre‐exercise PPT (or HPT). When interpreting the size of Pearson's correlation coefficients 0.90–1.00 is very strong, 0.70–0.90 is strong, 0.50–0.70 is moderate, and 0.30–0.50 is considered a weak correlation. (Mukaka, 2012)These values can be positive or negative to denote directionality (Mukaka, 2012).

## 3 RESULTS

### 3.1 Descriptives

A comprehensive analysis of a total sample of 28 participants was completed, demonstrated in Table 1. Two participants were removed from the analysis after completing the study as these individuals exceeded safety thresholds for pain threshold testing.

**TABLE 1:** Baseline demographic characteristics of the total sample.

Descriptives	Total sample (*n* = 28)
Age (years)	21 ± 4
Sex (*n* female)	21
Ethnicity (*n* Hispanic)	9
Race (*n*)	
White	22
Asian	3
African American	3
Height (in)	65.77 ± 3.69
Weight (kg)	64.75 ± 11.78
Minutes of exercise per week	242.50 ± 219.13
5 repetition maximum upper body (kg)	33.45 ± 15.54
5 repetition maximum lower body (kg)	52.72 ± 20.41

*Note*: Values reported represent the mean ± the standard deviation.

### 3.2 Immediate local and systemic changes in heat and pressure pain thresholds

#### 3.2.1 Heat pain threshold

The three‐way interaction effect of time, location, and condition was significant (F (1.71, 3.80) = 2.19, *p* = 0.04, *η* ^2^ *p* = 0.12). A significant increase in HPT was observed at the quadriceps during lower body resistance exercise postexercise (F (1, 4.49) = 2.55, *p* = 0.04) and at the deltoid during upper body resistance exercise postexercise (F (1, 11.88) = 12.31, *p* = 0.002). There were no additional significant effects found pre or post exercise at other testing sites.

**FIGURE 2:**
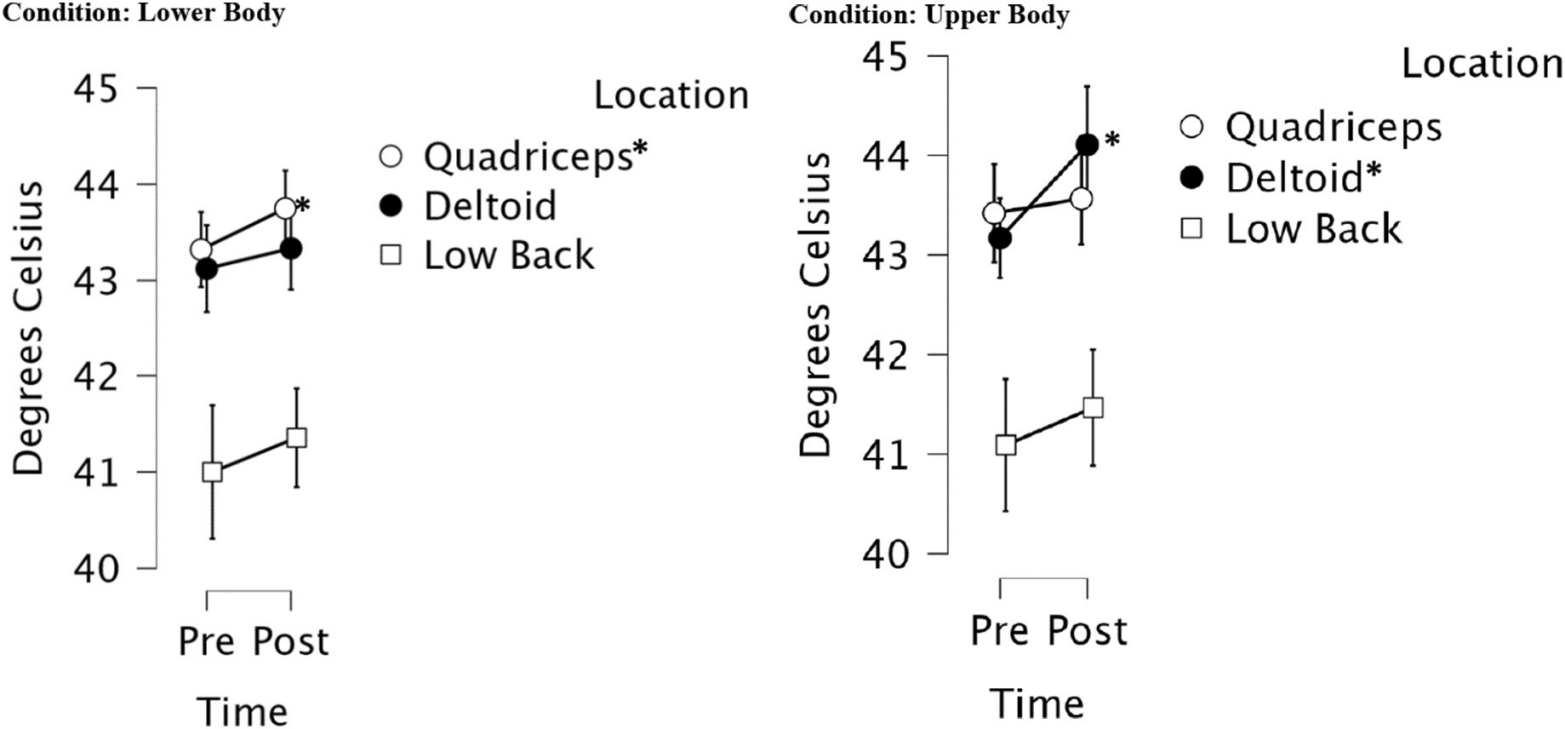
Heat pain threshold values pre and post exercise. Mean values for each site pre and post exercise were used. Error bars indicate a 95% confidence interval. Alpha level was set at *p* < 0.05 for statistical significance. * Indicates statistical significance.

Time × location interaction (F (1.86, 0.80) = 0.67, *p* = 0.45, *η* ^2^ *p* = 0.03) and location × condition (F (1.59, 1.52) = 1.87, *p* = 0.23, *η* ^2^ *p* = 0.05) were not significant. The main effect of location was found to be significant (F (1.60, 35.66) = 235.94, *p* < 0.001, *η* ^2^ *p* = 0.57) with HPT applied over the quadriceps displaying significantly higher thresholds compared the deltoid. Overall, the results suggest that HPT threshold values increased at the local exercising muscle postexercise as demonstrated in Figure 2.

#### 3.2.2 Pressure pain threshold

**FIGURE 3:**
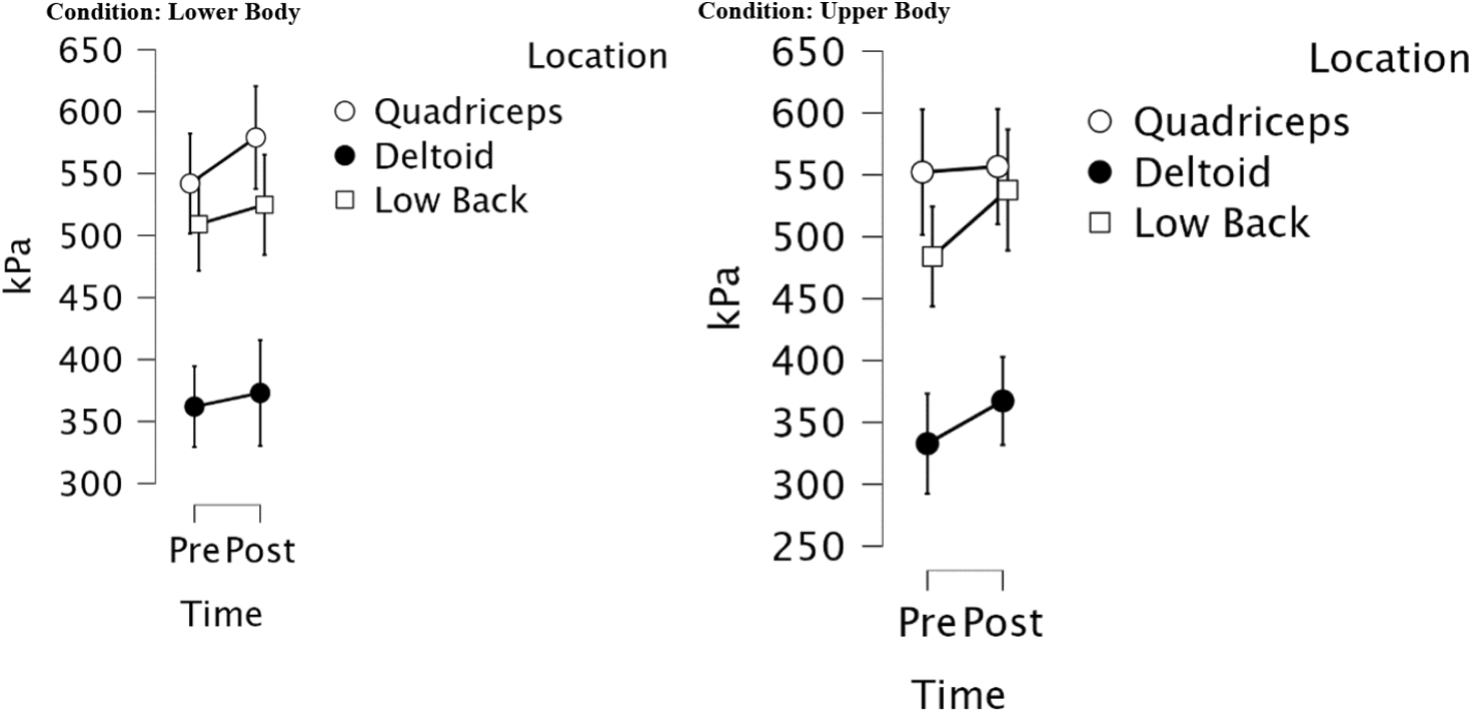
Pressure pain threshold values pre and post exercise. Mean values for each site pre and post exercise were used. Error bars indicate a 95% confidence interval. Alpha level was set at *p* < 0.05 for statistical significance.

A separate three‐way ANOVA analysis was performed with time (pre and post exercise) × location (quadriceps, deltoid, low back) × condition (upper or lower body) as repeated measures variables. The three‐way interaction effect of time, location, and condition was not significant ([1.744] = 1.948, *p* = 0.159, *η* ^2^ *p* = 0.070). Time × location (F(1.442) = 0.300, *p* = 0.670, *η* ^2^ *p* = 0.011) and condition × location was not significant (F(1.420) = 0.151, *p* = 0.786, *η* ^2^ *p* = 0.006). The main effect of location was significant (F(1.897) = 37.439, *p* < 0.001, *η* ^2^ *p* = 0.590) indicating PPT was significantly different at each testing site. Overall, the results suggest that location has a significant main effect on PPT, but the interaction effects of time × location, location × condition, and time × location × condition were not significant. There were no additional significant effects found pre or post exercise at other testing sites (*p*'s > 0.05). Results between upper and lower body can be seen in Figure 3.

Collectively, a local or systemic effect for pressure was not demonstrated during upper and lower body resistance exercise.

### 3.3 Correlations

CPM and TS were baseline tests that were measured to examine a potential association with EIH response. There were no significant effects found between any of these variables (*p*'s > 0.05). Table 2 displays Pearson's correlation coefficients and *p*‐values to assess the strength and significance among these variables.

**TABLE 2:** Correlations of EIH with QST.

Exercise	Variable		TS	CPM
Upper body	Quadriceps (°C)	Pearson's *r*	0.212	−0.199
		*p*‐value	0.279	0.309
	Deltoid (°C)	Pearson's *r*	0.088	0.099
		*p*‐value	0.657	0.616
	Lower back (°C)	Pearson's *r*	0.057	−0.237
		*p*‐value	0.772	0.224
	Quadriceps (kpa)	Pearson's *r*	0.154	0.034
		*p*‐value	0.442	0.865
	Deltoid (kpa)	Pearson's *r*	−0.181	−0.151
		*p*‐value	0.357	0.443
	Lower back (kpa)	Pearson's *r*	−0.216	0.188
		*p*‐value	0.270	0.338
Lower Body	Quadriceps (°C)	Pearson's *r*	−0.095	−0.021
		*p*‐value	0.630	0.916
	Deltoid (°C)	Pearson's *r*	−0.330	0.268
		*p*‐value	0.087	0.169
	Lower back (°C)	Pearson's *r*	0.103	−0.077
		*p*‐value	0.600	0.696
	Quadriceps (kpa)	Pearson's *r*	0.006	−0.207
		*p*‐value	0.976	0.301
	Deltoid (kpa)	Pearson's *r*	0.125	−0.250
		*p*‐value	0.526	0.199
	Lower back (kpa)	Pearson's *r*	0.243	−0.170
		*p*‐value	0.213	0.389

*Note*: Alpha level was set at *p* < 0.05 for statistical significance.

Abbreviation: EIH, exercise‐induced hypoalgesia.

* Indicates statistical significance *p*<0.05

## 4 DISCUSSION

The primary purpose of this investigation was to compare the effects of upper body or lower body exercise on immediate changes in pain sensitivity using a multi‐modal assessment (HPT and PPT) at multiple sites (deltoid, quadriceps, and low back). HPT, but not PPT, significantly increased at the local muscle site (deltoid, quadriceps) after upper body and lower body resistance exercise, respectively. While there were no significant changes during PPT testing or at any of the systemic muscle sites, EIH increased after resistance exercise with a medium effect size.

Consistent with prior literature, a significant local EIH effect was produced after resistance exercise. When examining a pain‐free population, hypoalgesia was present after PPT testing in the local exercising muscles following a moderate intensity upper body resistance exercise. (Lee, 2014) Another study performed by Keilman et al. (2017) demonstrated increased PPT of local muscle sites in the low back after performing kettlebell swings in 60 young, healthy participants. These studies provide evidence that EIH is present in the local exercising muscle when performing upper and lower body exercises. The results of the current study add to the body of literature by demonstrating this result with heat, further strengthening the notion that a single bout of resistance exercise elicits reductions in pain.

Although the EIH experienced in the exercising muscle is in agreement with prior literature, this increase in pain threshold was only seen after the noxious heat stimuli, not pressure. The perception of heat‐induced pain is subject to various influences, encompassing factors such as the mode of stimulus delivery, skin temperature, and stimulus duration. (Pertovaara et al., 1996; Price et al., 1992) The alterations in skin temperature have been observed to significantly impact the pain threshold for radiant heat stimuli, with lower skin temperatures associated with higher pain thresholds (Hall, 1955). However, the effect of both heat and pressure stimuli after dynamic resistance exercise not well known. One prior study investigated both stimuli in a healthy population and heat thresholds were not shown to increase. Only pressure pain tolerance was shown to increase, with no significant changes observed for HPT or PPT. (Vaegter et al., 2017) Although this is conflicting with the results from this study, the prior study only measured HPT at a systemic site after isometric exercise. Additional studies including a multimodal assessment method should be conducted before any definitive conclusions can be made about HPT after exercise.

Resistance exercise did not produce significant systemic EIH effects in our study. When examining EIH in systemic sites, the literature presents results that conflict with the current study's findings. In a study performed by Kotlyn et al. (2014), an increase in PPT was seen systemically immediately post resistance exercise. Although changes were observed 1 and 5 min post exercise, values returned to baseline after 15 min. (Focht & Koltyn, 2009; Koltyn & Arbogast, 1998) In agreement with the previously mentioned study, a study performed by McKean et al. (2018) demonstrated similar findings with values returning to baseline after 5 min. The results of these studies indicate the potential necessity of measuring pain thresholds of systemic sites immediately to observe EIH. It is possible PPT was not examined quickly enough after completing the exercise and, therefore, was not observed.

When assessing the limitations of this study, inadequate exertion during the 5‐RM assessment and subsequent exercise sessions might have influenced EIH. It is well established that the magnitude of EIH is contingent upon the intensity and duration of the exercise, with higher effort and intensity generally leading to more pronounced hypoalgesic effects. (Koltyn & Arbogast, 1998; Lee, 2014; Naugle et al., 2012; Vaegter & Jones, 2020) Furthermore, the limited total loads of dynamic resistance exercise in this study may have contributed to the absence of an increase in PPT. Typically, it takes over 30 min for a single bout of full‐body aerobic exercise to induce acute EIH responses, and around several minutes for isometric resistance exercise. (Koltyn, 2002). Thus, future studies may consider a full‐body approach, where different upper body or lower body exercises are combined to increase exercise duration and potentially elicit more robust EIH responses (Koltyn & Arbogast, 1998; Lee, 2014; Naugle et al., 2012; Vaegter & Jones, 2020). Another notable constraint was that the observed PPT values may not have exhibited statistically significant changes due to participants' challenges in accurately discerning the transition point from pressure to pain. Additionally, in this study HPT was examined prior to PPT, and the multimodal assessment may have also dampened the mechanical hypoalgesia effects. For future research, more closely monitoring participant exertion during the maximal repetition testing would be ideal.

## 5 CLINICAL IMPLICATIONS

This study informs the development of targeted rehabilitation strategies for pain management. Understanding localized and systemic effects of resistance exercise on pain perception allows clinicians to tailor exercise prescriptions for optimal pain relief. The results of this study have important implications for the development of future clinical trials as heat may be more sensitive to EIH effects during resistance exercise than pressure, suggesting a local C‐fiber mediated response. Variability in systemic effects highlight the need for individualized approaches in exercise interventions for pain relief, supporting the use of exercises local to the site of injury. Future studies should implement rehabilitation strategies utilizing dynamic resistance exercise in a clinical population to determine if the acute reductions in pain with exercise could contribute to long‐term reductions in chronic pain.

## 6 CONCLUSION

In summary, this study has significantly contributed to the existing literature on EIH by systematically investigating local and systemic muscular sites in a controlled manner. Our findings support prior research, demonstrating the robust local effects of EIH. (Keilman et al., 2017; Lee, 2014; McKean et al., 2018) Future research should aim to further elucidate the underlying mechanisms of EIH and explore individualized approaches to pain management and rehabilitation. Ultimately, this research encourages the continued refinement of exercise interventions for pain management, with the aim of optimizing their effectiveness and enhancing the quality of life for those experiencing pain.

## FUNDING INFORMATION

The authors did not receive support from any organization for the submitted work. The authors have no relevant financial or non‐financial interests to disclose.

## CONFLICT OF INTEREST STATEMENT

The authors report there are no competing interests to declare.

## ETHICS STATEMENT

All procedures performed in studies involving human participants were in accordance with the ethical standards of the institutional research committee and with the 1964 Helsinki Declaration and its later amendments or comparable ethical standards. This study was approved by the University of Central Florida Institutional Review Board for Human Subjects Research.

## PATIENT CONSENT STATEMENT

All participants provided written informed consent to enroll in the study. Public Trials Registry: NCT05985382. IRB Study #5752.

## Data Availability

Data set available upon request.
